# Systematic Evaluation of Structure-Activity Relationships of the Riminophenazine Class and Discovery of a C2 Pyridylamino Series for the Treatment of Multidrug-Resistant Tuberculosis

**DOI:** 10.3390/molecules17044545

**Published:** 2012-04-17

**Authors:** Binna Liu, Kai Liu, Yu Lu, Dongfeng Zhang, Tianming Yang, Xuan Li, Chen Ma, Meiqin Zheng, Bin Wang, Gang Zhang, Fei Wang, Zhenkun Ma, Chun Li, Haihong Huang, Dali Yin

**Affiliations:** 1 State Key Laboratory of Bioactive Substances and Function of Natural Medicine & Beijing Key Laboratory of Active Substance Discovery and Druggability Evaluation, Institute of Materia Medica, Peking Union Medical College and Chinese Academy of Medical Sciences, 1 Xian Nong Tan Street, Beijing 100050, China; 2 Beijing Tuberculosis and Thoracic Tumor Research Institute, 97 Ma Chang Street, Beijing 101149, China; 3 Global Alliance for TB Drug Development, 40 Wall Street, New York, NY 10005, USA

**Keywords:** anti-tuberculosis, MDR-TB, riminophenazine, clofazimine, skin pigmentation

## Abstract

Clofazimine, a member of the riminophenazine class of drugs, is the cornerstone agent for the treatment of leprosy. This agent is currently being studied in clinical trials for the treatment of multidrug-resistant tuberculosis to address the urgent need for new drugs that can overcome existing and emerging drug resistance. However, the use of clofazimine in tuberculosis treatment is hampered by its high lipophilicity and skin pigmentation side effects. To identify a new generation of riminophenazines that is less lipophilic and skin staining, while maintaining efficacy, we have performed a systematic structure-activity relationship (SAR) investigation by synthesizing a variety of analogs of clofazimine and evaluating their anti-tuberculosis activity. The study reveals that the central tricyclic phenazine system and the pendant aromatic rings are important for anti-tuberculosis activity. However, the phenyl groups attached to the C2 and N5 position of clofazimine can be replaced by a pyridyl group to provide analogs with improved physicochemical properties and pharmacokinetic characteristics. Replacement of the phenyl group attached to the C2 position by a pyridyl group has led to a promising new series of compounds with improved physicochemical properties, improved anti-tuberculosis potency, and reduced pigmentation potential.

## 1. Introduction

Tuberculosis (TB) infects about some 9.2 million and kills approximately 1.8 million people globally every year [1]. Despite the best treatment and control efforts using available vaccines and drugs, the prevalence of TB throughout the World remains high, due in part to the emergence of multidrug resistant *Mycobacterium tuberculosis* (MDR-TB) [2] and the global HIV epidemic. The treatment of MDR-TB and TB/HIV co-infection using the currently available drug regimens is extremely challenging [3]. New and more effective drugs are needed to address these challenges. 

**Figure 1:**
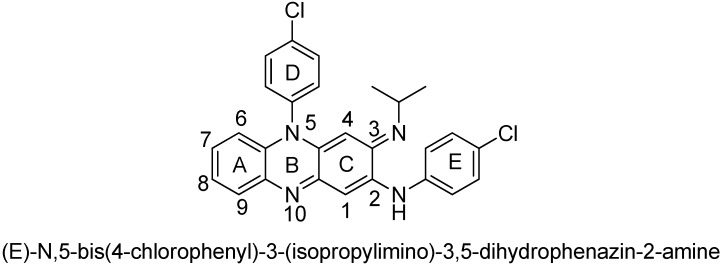
Structure of clofazimine.

In an effort to identify new anti-tuberculosis agents that can be effective against MDR-TB, we decided to revisit the riminophenazine class of drugs. Clofazimine (CFZ, Figure 1), a member of the riminophenazine class, is the cornerstone agent for the treatment of leprosy. This agent was first synthesized by Barry’s group [4] and has demonstrated potent *in vitro* activity against *M. tuberculosis* [5]. Recent studies indicate that clofazimine is equally potent against drug-susceptible and drug-resistant (including MDR) *M. tuberculosis* strains, which suggests that it operates via a novel mechanism of action. In addition, the frequency of resistance development for this compound is extremely low as compared with other anti-tuberculosis drugs [6]. Clofazimine is currently in clinical trials for the treatment of MDR-TB to address the urgent need for new drugs that can overcome existing and emerging drug resistance in tuberculosis. However, the widespread use of clofazimine in tuberculosis treatment is hampered by its extremely high lipophilicity and strong skin pigmentation side effects. Although a large number of analogues of clofazimine have been prepared, they are limited in scope. The modifications are limited to the substituents on two pendant phenyls and the imino group [7]. To identify a new generation of riminophenazines that is both less lipophilic and skin staining, while maintaining its efficacy against tuberculosis, a systematic structure-activity and structure-liability relationship (SLR) study is needed.

Clofazimine is a lipophilic molecule as indicated by its high Clog P value of approximately 7.48 [8]. Its undesirable physicochemical and pharmacokinetic properties contribute to its side effects and limit its clinical use. In particular, its extremely long half-life and propensity for tissue accumulation and precipitation together with the dye property lead to unwelcome skin pigmentation. Herein we report our SAR and SLR studies on the riminophenazine class with a goal of identifying a new generation of compounds with improved physicochemical properties that are highly efficacious against both drug-susceptible and MDR-TB and at the same time incur reduced skin pigmentation potential.

## 2. Results and Discussion

**Scheme 1:**
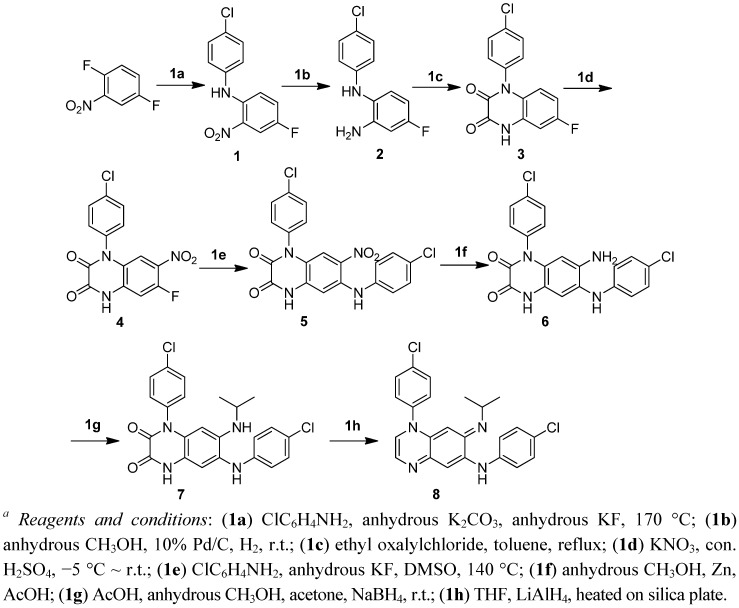
Synthesis of A-ring deletion compound, riminoquinoxaline. *^a^*

**Scheme 2:**
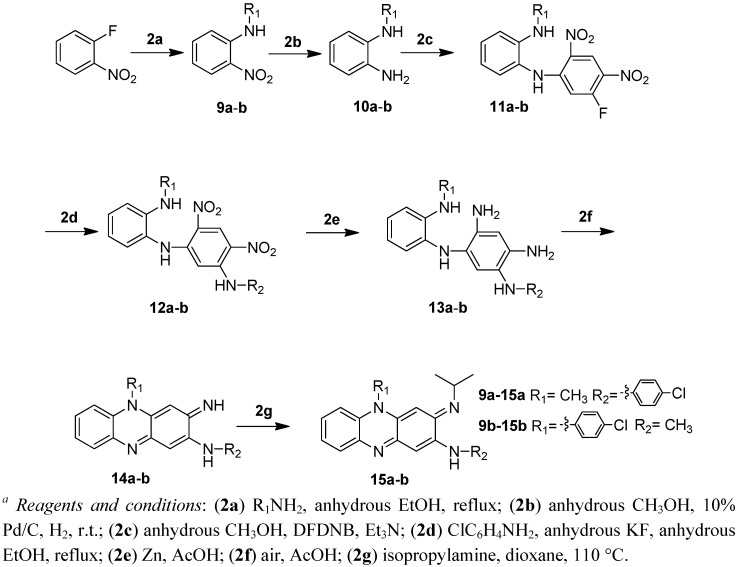
Synthesis of D-ring deletion riminophenazines. *^a^*

To improve its physicochemical properties and circumvent the skin pigmentation problem of clofazimine, we first explored the possibility of decreasing the intrinsic colour of the molecules by reducing the conjugation system of the riminophenazine core structure. Thus, we systematically removed one of the phenyl rings (A, D or E) from the molecule while keeping rings B, C, and the imino moiety intact. The syntheses of these analogs are illustrated in Scheme 1 and Scheme 2, respectively. 

The synthesis of the A-ring deletion analogs (riminoquinoxalines) was started from 2,5-difluoronitrobenzene (Scheme 1). After replacement of the *ortho*-fluoro group with 4-chloroaniline, compound **1** was reduced to give diamine **2**. Compound **2** was then treated with ethyl oxalyl chloride to form the B ring. After nitration of **3**, the activated fluoro at the *ortho* position of the nitro adduct of **4** was replaced by 4-chloroaniline. Reduction of the nitro group in **5** produced compound **6**, followed by reductive alkylation to provide compound **7**. Reduction of the carbonyl groups in **7** with LiAlH_4_ followed by an air auto-oxidization provided the target compound **8**.

Both D-ring and E-ring deletion compounds **15a** and **15b** were synthesized starting from 1,5-difluoro-2,4-dinitrobenzene (DFDNB) (Scheme 2). 

Thus compound **9a**–**b** was reduced to diamine **10a**–**b** which was then coupled with 1,5-difluoro-2,4-dinitrobenzene to give **11a**–**b**. The second fluorine atom was replaced by 4-chloroaniline to afford **12a**–**b**. Reduction of both nitro groups gave **13a**–**b** which underwent spontaneous cyclization to afford riminophenazines **14a**–**b**. Replacement of the imine with isopropylamine gave the target compounds **15a**–**b**.

The A-ring deletion compound **8** exhibited considerably reduced *in vitro* activity against *M tuberculosis* (MIC_90_ = 18.89 μM, Table 1). When the appendant phenyl group D or E was replaced by a methyl group (compounds **15a** and **15b**), the antimycobacterial activity was also abolished (MIC_90_ ≥ 42.45 μM and MIC_90_ = 21.23 μM respectively, Table 1). 

**Table 1:** *In vitro* anti-tuberculosis activity of the riminophenazine analogues with systematic deletion of one of the phenyl rings against *M. tuberculosis* H_37_Rv.

Comp.	Structure	MIC_90_ (μM)	Comp.	Structure	MIC_90_ (μM)
**CFZ**	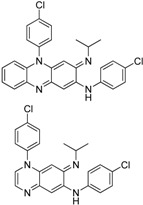	0.25	**15a**	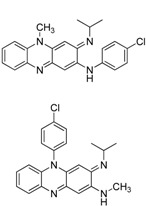	>42.45
**8**		18.89	**15b**		21.23

**Scheme 3:**
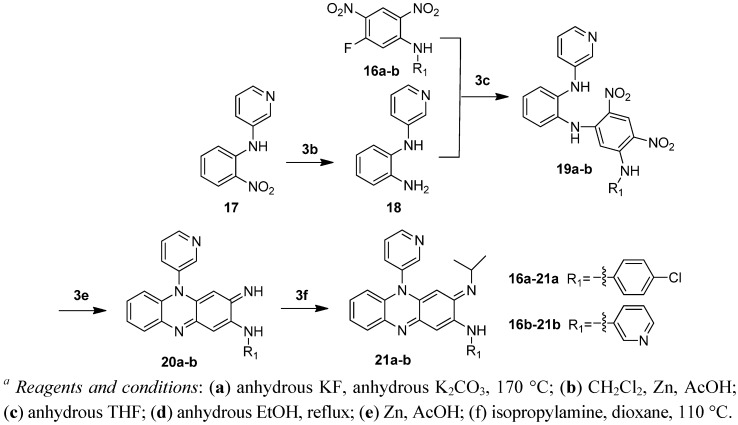
Synthesis of compound **21a**–**b**. *^a^*

**Scheme 4:**
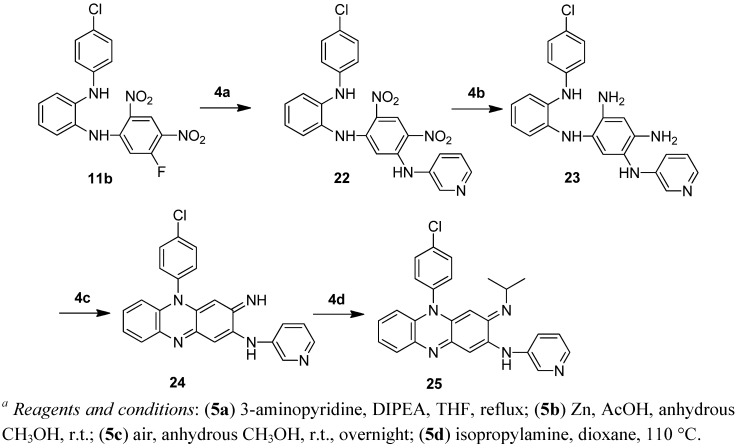
Synthesis of compound **25**. *^a^*

Failing to identify analogues that retained antimycobacterial activity through dissecting their core structures, we turned our attention to the modification of the D- and E-rings of the molecule to improve its physicochemical properties and thereby change tissue distribution and tissue accumulation. It was anticipated that the Clog P of the molecule would decrease if one or both of the phenyl groups were replaced by a pyridyl group. Thus compounds **21a**–**b** and **25** were synthesized following a modified synthetic procedure (Scheme 3 and Scheme 4). 

The Clog P of the molecule decreases by 2 if one of the phenyl groups is replaced by a pyridyl group and by 4 if both phenyl groups are replaced by pyridine. In the *in vitro* assay, the N5 pyridyl analogues **21a** and **21b** showed reduced potency (MIC_90_ 0.57 and 1.23 μM respectively). To our surprise, the C2 pyridylamino analogue **25** demonstrated improved potency against *M tuberculosis* H_37_Rv (MIC_90_ 0.07 μM) as compared to clofazimine (MIC_90_ 0.25 μM) (Table 2). 

**Table 2:** Riminophenazines with R_1_ and R_3_ variation and their anti-tuberculosis activity against *M. tuberculosis* H_37_Rv, physicochemical property (log P). 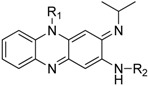

Comp.	R_1_	R_2_	ClogP	logP	MIC_90_ (μM)
**CFZ**	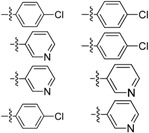		7.137	5.43	0.25
**21a**			5.322	-	0.57
**21b**			3.507	2.76	1.23
**25**			5.322	3.43	0.07

In addition, compound **25** demonstrated excellent *in vivo* efficacy in a mouse tuberculosis model as measured by a significant drop in bacterial colony-forming units (CFU) in the lung as compared to a no-treatment control group [9]. The mean log CFU count in the lung dropped by about 5 units (3.83 ± 0.27 CFU/lung ) for the group treated with compound **25** as compared to the untreated group (8.53 ± 0.32 CFU/lung), and by about 3 units as compared to rifampin (RIF)-treated positive control groups (RIF 6.71 ± 0.13 CFU/lung, CFZ 6.33 ± 0.07 CFU/lung). Compound **25** also demonstrated a shorter half-life (t_1/2_) as compared to clofazimine and reduced abdominal tissue pigmentation, which is strikingly different from the effect on those animals treated by clofazimine (data not shown). This SAR and SLR study illustrates that all five aromatic rings of clofazimine are important for maintaining potent anti-tuberculosis activity. The two pendant phenyl rings attached to the C2 and N5 positions, especially the C2 position, can be replaced by a pyridyl group, and such analogues have demonstrated potent *in vitro* anti-tubercular activity, excellent *in vivo* efficacy, and reduced skin pigmentation potential. 

## 3. Experimental

### 3.1. Reagents and Instrumentation

Unless otherwise indicated, all reagents and solvents were used as received from the suppliers. Reactions were monitored for completion by thin layer chromatography (TLC) using silica gel GF-254 plates with detection under UV (254 nm) light. ^1^H-NMR spectra were recorded on a Varian 300 MHz or 400 MHz instrument in CDCl_3_, CD_3_OD, DMSO-*d*_6_ or acetone-*d_6_* solutions. ^13^C-NMR spectra were recorded on a Varian instrument at 100 MHz or 150 MHz with CDCl_3_ or DMSO-*d*_6_ as solvents. Chemical shifts are reported in parts per million (*δ*) downfield from tetramethylsilane (TMS). Coupling constants (*J*) are reported in Hz. High-resolution mass spectra were acquired from an Agilent 1100 series LC/MSD mass spectrometer. All MS experiments were performed using electrospray ionization (ESI-TOF^+^) in positive ion mode. Column chromatography was carried out with silica gel (200~300 mesh).

### 3.2. Chemistry

#### 3.2.1. Synthesis of *2-(4-chloroanilino)-5-fluoronitrobenzene* (**1**)

4-Chloroaniline (12.76 g, 100 mmol), 2,5-difluoronitrobenzene (8.0 g, 50 mmol), anhydrous K_2_CO_3_ (3.5 g, 25 mmol) and anhydrous KF (2.9 g, 50 mmol) were mixed and heated at 170 °C for 14 h. After cooling to room temperature, water was added and the solid formed was filtered and washed with water. The crude product was purified via recrystallization from 95% ethanol to give **1** as an orange solid (11.8 g, 89%). ^1^H-NMR (300 MHz, CDCl_3_) *δ* 9.25 (s, 1H), 7.94–7.90 (m, 1H), 7.39 (d, *J* = 8.7 Hz, 2H), 7.21–7.17 (m, 4H). ESI/MS (*m/z*): 267 [M+H]^+^.

#### 3.2.2. Synthesis of *1-(4-chlorophenyl)-6-fluoroquinoxaline-2,3(1*H,*4*H*)-dione* (**3**)

Compound **1** above (6.45 g, 24 mmol) was suspended in anhydrous methanol (100 mL). The mixture was hydrogenated with 10% Pd/C (1.3 g) at room temperature at 1 atmosphere pressure of hydrogen for 8 h. After removal of catalyst, the solvent was concentrated under reduced pressure. The residue was dissolved in toluene (150 mL) and ethyl oxalyl chloride (10 mL, 89 mmol) was added. The mixture was refluxed for 1 h and then cooled to room temperature Approximately one half of the solvent was removed under reduced pressure and the resulting solid was filtered and recrystallized from methanol to give **3** as a white solid (4.97 g, 71%). ^1^H-NMR (300 MHz, DMSO-*d_6_*) *δ* 12.18 (s, 1H), 7.69 (d, *J* = 8.7 Hz, 2H), 7.43 (d, *J* = 8.7 Hz, 2H), 6.99 (dd, *J* = 9.0, 2.7 Hz, 1H), 6.87–9.80 (m, 1H), 6.34 (dd, *J* = 9.3, 5.1 Hz, 1H). ESI/MS (*m/z*): 291 [M+H]^+^.

#### 3.2.3. Synthesis of *1-(4-chlorophenyl)-6-fluoro-7-nitroquinoxaline-2,3(1*H,*4*H*)-dione* (**4**)

Compound **3** above (2.9 g, 10 mmol) was dissolved in concentrated sulfuric acid (30 mL) and cooled to −5 °C. KNO_3_ (1.1 g, 8 mmol) was added into the solution portion-wise. After addition, the mixture was stirred at 0 °C for 1 h and then at room temperature for 1 h. The reaction mixture was slowly poured into ice water, and the solid formed was filtered and washed with water. The product was air-dried to give a light yellow solid 3.4 g (quantitative yield). ^1^H-NMR (300 MHz, DMSO-*d_6_*) *δ* 12.62 (s, 1H), 7.75 (d, *J* = 8.7 Hz, 2H), 7.45 (d, *J* = 8.7 Hz, 2H), 7.19 (d, *J* = 11.7 Hz, 1H), 6.96 (d, *J* = 6.9 Hz, 1H). ESI/MS (*m/z*): 336 [M+H]^+^.

#### 3.2.4. Synthesis of *1-(4-chlorophenyl)-6-(4-chloroanilino)-7-nitroquinoxaline-2,3(1*H,*4*H*)-dione* (**5**)

Compound **4** above (0.34 g, 1.0 mmol), DMSO (3 mL), anhydrous KF (0.12 g, 2.0 mmol), and 4-chloroaniline (1.27 g, 10 mmol) were heated at 140 °C for 25 h. After cooling to room temperature, 5 M HCl was added and the resulting solid was collected by filtration and washed with water. The crude product was purified by column chromatography to give **5** as an orange solid (0.22 g, 50%). ^1^H-NMR (300 MHz, DMSO-*d_6_*) *δ* 12.09 (s, 1H), 9.35 (s, 1H), 7.76 (d, *J* = 8.4 Hz, 2H), 7.52–7.46 (m, 4H), 7.35 (d, *J* = 8.4 Hz, 2H), 7.01 (s, 1H), 6.99 (s, 1H). ESI/MS (*m/z*): 443 [M+H]^+^.

#### 3.2.5. Synthesis of *1-(4-chlorophenyl)-6-(4-chloroanilino)-7-(isopropylamino)-quinoxaline-2,3-(1*H,*4*H*)-dione* (**7**)

Zinc powder (0.13 g) was added portionwise to a vigorously stirred mixture of **5** (0.08 g, 0.18 mmol) in glacial acetic acid (2 mL) and anhydrous methanol (5 mL). After the reaction was complete, as confirmed by TLC, the reaction mixture was filtered. The filtrate was concentrated under reduced pressure. Water was then added to the residue. After filtration, the yellow solid was dissolved in a mixture of glacial acetic acid (4 mL) and acetone (0.3 mL) in anhydrous methanol (2 mL). The resulting mixture was stirred at room temperature for 30 min., and then sodium borohydride (76 mg, 2.0 mmol) was added and stirred for 30 min. The reaction mixture was concentrated under reduced pressure and water was added to the residue. The resulting solid was collected by filtration, washed with water, and dried. The crude product was purified by column chromatography to give **7** as a solid (30 mg, 37%). ^1^H-NMR (300 MHz, acetone-*d_6_*) *δ* 7.70 (d, *J* = 8.7 Hz, 2H), 7.51 (d, *J* = 8.7 Hz, 2H), 7.15 (d, *J* = 8.7 Hz, 2H), 7.12 (s, 1H), 6.75 (d, *J* = 8.7 Hz, 2H), 5.76 (s, 1H), 3.18 (m, 1H), 0.99 (d, *J* = 6.3 Hz, 6H). ESI/MS (*m/z*): 445 [M+H]^+^. 

#### 3.2.6. Synthesis of *1-(4-chlorophenyl)-6-(4-chloroanilino)-7-isopropylimino-1,7-dihydroquinoxaline* (**8**)

Lithium aluminum hydride (8 mg, 0.21 mmol) was added into a mixture of **7** (30 mg, 0.07 mmol) in THF (5 mL). The mixture was heated at 50 °C for 1 h under nitrogen atmosphere. After cooling to room temperature, 3 drops of ethyl acetate were added. The mixture was filtered and washed with ethyl acetate. The filtrate was washed with brine. The organic layer was separated and applied to preparative TLC. The TLC plate was heated in an oven (100 °C) for 30 min. before being developed with CHCl_3_/MeOH (20:1). The red band (R_f_: 0.34) was collected and washed with anhydrous methanol. The washing was concentrated, and the residue was further purified by column chromatography (HL20, eluted with MeOH) to give 6 mg of **8** as a red solid (21%). ^1^H-NMR (300 MHz, CD_3_OD) *δ* 8.62 (d, *J* = 3.6 Hz, 1H) 8.28 (d, *J* = 3.6 Hz, 1H) 7.78 (d, *J* = 8.7 Hz, 2H), 7.70 (d, *J* = 9.0 Hz, 2H), 7.49 (s, 1H), 7.42 (d, *J* = 8.7 Hz, 2H), 7.31 (d, *J* = 9.0 Hz, 2H), 6.22 (s, 1H), 3.65–3.61 (m, 1H), 1.23 (d, *J* = 6.3 Hz, 6H). ^13^C-NMR (150 MHz, CD_3_OD) *δ* 149.3, 146.9, 142.6, 140.1, 139.5, 138.9, 134.3, 132.1, 131.2, 131.0, 129.0, 125.1, 108.6, 92.1, 46.9, 21.4. HRMS (ESI-TOF^+^): *m/z* [M+H]^+^ calcd. for C_23_H_21_Cl_2_N_4_: 423.1137; found: 423.1141.

#### 3.2.7. Synthesis of *2-methylamino-nitrobenzene* (**9a**)

2-Fluoronitrobenzene (2.82 g, 20 mmol) was dissolved in anhydrous ethanol (20 mL), and 33% aqueous methylamine solution (4.6 mL) was added. The mixture was heated to reflux until complete consumption of 2-fluoronitrobenzene. After cooling to room temperature, the solid formed was filtered and washed with 95% ethanol and dried to give the title compound as a red oil (quantitative yield). ^1^H-NMR (400 MHz, acetone-*d_6_*) *δ* 8.10 (d, *J* = 8.0 Hz, 1H), 7.54 (t, *J* = 7.2, 8.4 Hz, 1H), 7.01 (d, *J* = 8.4 Hz, 1H), 6.68 (t, *J* = 7.2, 8.0 Hz, 1H), 3.07 (d, *J* = 5.2 Hz, 3H). ESI/MS (*m/z*): 153 [M+H]^+^.

#### 3.2.8. Synthesis of N*-(4-chlorophenyl)-2-nitroaniline* (**9b**)

2-Fluoronitrobenzene (14.1 g, 100 mmol), 4-chloroaniline (19.1 g, 150 mmol), anhydrous KF (5.8 g, 100 mmol) and anhydrous K_2_CO_3_ (13.8 g, 100 mmol) were heated at 160 °C for 10 h. The reaction mixture was cooled and water and ethyl acetate were added. The aqueous layer was extracted with ethyl acetate. The organic layer was combined and washed with 2 N HCl and dried over anhydrous Na_2_SO_4_. The solvent was concentrated under reduced pressure and the residue was recrystallized with anhydrous ethanol to give the title compound as red solid (20 g, 80%). ^1^H-NMR (300 MHz, CDCl_3_) *δ* 9.40 (s, 1H), 8.23–8.19 (d, *J* = 8.7 Hz, 1H), 7.41–7.37 (m, 3H), 7.23–7.16 (m, 3H), 6.78 (t, *J* = 8.4, 8.1 Hz, 1H).

#### 3.2.9. Synthesis of *1-methylamino-2-(5-fluoro-2,4-dinitroanillino)-benzene* (**11a**)

A suspension of **9a** (1.1 g, 5.0 mmol) in anhydrous methanol (20 mL) was hydrogenated with 10% Pd/C (0.06g) under atmospheric pressure until the reaction mixture turned colourless. After filtration, the filtrate was treated with DFDNB (1.03 g, 5.0 mmol) and Et_3_N (0.7 mL, 5.0 mmol) and stirred at room temperature for 1 h. The solid formed was filtered and washed with methanol to give yellow solid (78%). ^1^H-NMR (300 MHz, acetone-*d_6_*) *δ* 9.74 (s, 1H), 9.04–9.02 (d, *J* = 7.8 Hz, 1H), 7.34–7.29 (t, *J* = 7.5 Hz, 1H), 7.20–7.17 (d, *J* = 7.5 Hz, 1H), 6.81–6.76 (d, *J* = 7.5 Hz, 1H), 6.74–6.71 (t, *J* = 7.8 Hz, 1H), 6.09 (d, *J* = 14.1 Hz, 1H), 5.13 (d, *J* = 3.9 Hz, 1H), 2.80–2.78 (d, *J* = 5.1 Hz, 3H). ESI/MS (*m/z*): 307 [M+H]^+^.

#### 3.2.10. Synthesis of *1-(4-chloroanilino)-2-(5-fluoro-2,4-dinitroanilino)benzene* (**11b**)

Zinc powder (9 g) was added portionwise into a mixture of **9b** (2.5 g, 10 mmol) in CH_2_Cl_2_ (50 mL) and glacial acetic acid (3 mL) and stirred at room temperature for 4 h. After filtration, the filtrate was concentrated, and the residue was treated with DFDNB (2.04 g, 10 mmol) and Et_3_N (1.01 g, 10 mmol) in anhydrous methanol (100 mL). The mixture was refluxed for 5 h. and then cooled to room temperature. The solid formed was filtered, washed with ethanol and dried to give the title compound as a red solid (2.60 g, 65%). M.p. 216–217 °C. ^1^H-NMR (300 MHz, DMSO-*d*_6_) *δ* 9.96 (s, 1H), 8.87 (d, *J* = 8.1 Hz, 1H), 7.87 (s, 1H), 7.35–7.30 (m, 3H), 7.22–7.19 (d, *J* = 9.0 Hz, 2H), 7.07–7.02 (m, 1H), 6.97–6.94 (d, *J* = 9.0 Hz, 2H), 6.46 (d, *J* = 14.4 Hz, 1H). ESI/MS (*m/z*): 403 [M+H]^+^.

#### 3.2.11. Synthesis of *1-[5-(4-chloroanilino)-2,4-dinitroanilino]-2-methylaminobenzene* (**12a**)

A stirred suspension of **11a** (1.34 g, 3.6 mmol), 4-chloroanilline (0.92 g, 7.2 mmol), and anhydrous KF (0.21 g, 3.6 mmol) in anhydrous ethanol (30 mL) was refluxed for 4 h. After being cooled to room temperature, the solid formed was filtered and washed with anhydrous ethanol to give the title compound as an orange solid (1.52 g, 88%). ^1^H-NMR (300 MHz, DMSO-*d_6_*) *δ* 9.48 (brs, 2H), 9.00 (s, 1H), 7.30 (d, *J* = 8.7 Hz, 2H), 7.19 (d, *J* = 9.0 Hz, 2H), 7.08 (t, *J* = 7.2 Hz, 1H), 6.99 (dd, *J* = 7.5, 1.5 Hz, 1H), 6.70 (d, *J* = 7.8 Hz, 1H), 6.56 (t, *J* = 7.5 Hz, 1H), 6.00 (s, 1H), 4.85 (d, *J* = 8.7 Hz, 1H), 3.23–3.20 (m, 1H), 1.80–1.76 (m, 2H), 1.68–1.56 (m, 3H), 1.36–1.24 (m, 2H), 1.12–1.01 (m, 3H).

#### 3.2.12. Synthesis of *1-[5-methylamino-2,4-dinitroanilino]-2-(4-chlorophenyl)benzene* (**12b**)

A mixture of **11b** (1.05 g, 2.6 mmol), 30% methylamine in ethanol (5 mL, 48.4 mmol) in anhydrous ethanol (100 mL) was refluxed for 4 h. The reaction mixture was cooled to room temperature and filtered. The solid obtained was washed with ethanol and dried to give the title compound as a red solid (1.0 g, 93%). It was used directly in the next step without further purification. M.p. 239–240 °C. ^1^H-NMR (300 MHz, DMSO-*d*_6_) *δ* 9.56 (s, 1H), 8.96 (s, 1H), 8.39 (d, *J* = 4.8 Hz, 1H), 7.87(s, 1H), 7.41 (d, *J* = 6.9 Hz, 1H), 7.34–7.24 (m, 2H), 7.19 (d, *J* = 9.0 Hz, 2H), 7.08–7.02 (m, 1H), 6.95 (d, *J* = 9.0 Hz, 2H), 5.71 (s, 1H), 2.65 (d, *J* = 5.1 Hz, 3H). ESI/MS (*m/z*): 436 [M+Na]^+^.

#### 3.2.13. Synthesis of *2-(4-chloroanilino)-5-methyl-3-isopropylimino-3,5-dihydrophenazine* (**15a**)

Zinc powder (1.96 g) was added portionwise to a suspension of **12a** (0.48 g, 1.0 mmol) in glacial acetic acid (10 mL) and heated at 50 °C for 30 min. After filtration, the filtrate was stirred in contact with air overnight. The reaction mixture was concentrated under reduced pressure and adjusted to alkaline with ammonia, and then the solid formed was filtered and washed with water to give a black solid 0.34 g. The black solid was taken up in dioxane (5 mL) and isopropylamine (2.2 mL, 25.7 mmol) was added. The mixture was heated in a sealed bomb at 110 °C for 7 h. After being cooled to room temperature, water was added to the mixture and the solid formed was filtered and purified by column chromatography (eluted with P.E./ethyl acetate: 4/1 to 2/1 to 100% ethyl acetate) to give 0.13 g of the title compound (29%). M.p. 98–102 °C. ^1^H-NMR (300 MHz, CDCl_3_) *δ* 7.65 (dd, *J* = 7.8, 1.5 Hz, 2H), 7.44 (d, *J* = 8.4 Hz, 1H), 7.32–7.31 (m, 1H), 7.30 (s, 4 H), 7.19–7.14 (m, 1H), 6.78 (s, 1H), 6.17 (s, 1H), 4.39 (m, 1H), 3.92–3.88 (m, 1H), 2.58–2.52 (m, 2H), 2.07–1.85 (m, 5 H), 1.56–1.51 (m, 2H), 1.35–1.34 (m, 1H), 1.31 (d, *J* = 6.3 Hz, 6H). ^13^C-NMR (100 MHz, CDCl_3_) *δ* 152.3, 150.6, 143.1, 138.8, 137.0, 133.7, 131.2, 129.2, 128.6, 127.8, 126.9, 122.5, 122.4, 114.2, 99.7, 90.5, 61.2, 49.7, 28.6, 26.5, 25.7, 23.9. HRMS (ESI-TOF^+^): *m/z* [M+H]^+^ calcd. for C_27_H_30_ClN_4_: 445.2159; found: 445.2161.

#### 3.2.14. Synthesis of *2-methylamino-5-(4-chlorophenyl)-3-imino-3,5-dihydrophenazine* (**14b**)

Zinc powder (4.0 g, 61.5 mmol) was added portionwise to a suspension of **12b** (0.8 g, 1.9 mmol) in glacial acetic acid (80 Ml) and stirred for 4 h. The reaction mixture was filtered and washed with glacial acetic acid. The filtrate was stirred in contact with air overnight. The solvent was concentrated and the residue was treated with water and adjusted to alkaline with ammonia. The solid formed was filtered, washed with water and dried. The crude solid was purified by neutral aluminum oxide (100–200 mesh) column chromatography eluted with ethyl acetate/methanol (10:1 to 5:1) to give the title compound as a dark red solid (0.28 g, 43%). M.p. 218–220 °C. ^1^H-NMR (300 MHz, DMSO-*d*_6_) *δ* 9.20 (s, 1H), 7.83 (d, *J* = 8.7 Hz, 2H), 7.65 (d, *J* = 5.1 Hz, 1H), 7.55 (d, *J* = 8.4 Hz, 2H), 7.24–7.21 (m, 2H), 6.95 (brs, 1H), 6.48 (d, *J* = 8.4 Hz, 1H), 6.10 (s, 1H), 5.31 (s, 1H), 2.88 (d, *J* = 2.7 Hz, 3H). ESI/MS (*m/z*): 335 [M+H]^+^.

#### 3.2.15. Synthesis of *2-methylamino-3-isopropylimino-5-(4-chlorophenyl)-3,5-dihydro-phenazine* (**15b**)

A mixture of **14b** (0.15 g, 0.45 mmol), isopropylamine (1 mL, 12.0 mmol) and dioxane (20 mL) was heated in a sealed tube at 120 °C for 10 h. After being cooled to room temperature, the reaction mixture was concentrated under reduced pressure. The residue was purified by neutral aluminum oxide (100–200 mesh) column chromatography eluted with hexane/ethyl acetate (2:1) to give 150 mg of **15b** as a red solid (88%). M.p. 235–237 °C. ^1^H-NMR (300MHz, acetone-*d*_6_) *δ* 7.85–7.82 (d, *J* = 8.7 Hz, 2H), 7.61–7.58 (d, *J* = 9.3 Hz, 1H), 7.54–7.51 (d, *J* = 8.4 Hz, 2H), 7.18–7.10 (m, 2H), 6.95 (brs, 1H), 6.48–6.45 (d, *J* = 9.0 Hz, 1H), 5.99 (s, 1H), 5.24 (s, 1H), 3.44–3.36 (m, 1H), 2.96 (d, *J* = 2.7 Hz, 3H), 0.98 (d, *J* = 6.0 Hz, 6H). ^13^C-NMR (125 MHz, CDCl_3_) *δ* 151.1, 150.8, 149.2, 136.4, 135.7, 135.5, 134.7, 131.5, 131.1, 130.5, 127.7, 126.5, 122.7, 113.7, 95.2, 88.9, 49.2, 29.2, 23.5. HRMS (ESI-TOF^+^): *m/z* [M+H]^+^ calcd. for C_22_H_22_ClN_4_: 377.1527; found: 377.1510.

#### 3.2.16. N1*-(5-((4-chlorophenyl)amino)-2,4-dinitrophenyl)-*N2*-(pyridin-3-yl)benzene-1,2-diamine* (**19a**)

Compound **17** (0.215g, 1.0 mmol) was suspended in anhydrous methanol (10 mL). Pd/C (10%, 40 mg) was added and the mixture was hydrogenated under atmospheric pressure until the reaction mixture turned colourless. The Pd/C was filtered off. The filtrate was concentrated *in vacuo* to give a light yellow oil. The oil was dissolved in THF (10 mL) and to this solution **16a** (0.312 g 1.0 mmol) and diisopropylethylamine (0.17 mL, 1.0 mmol) were added. The mixture was refluxed for 20 h and then the reaction mixture was cooled to room temperature and concentrated *in vacuo*. The residue was added with water and filtered. The crude product was purified via chromatography (PE/EA: 2/1 to 1/1) to give the title compound as a yellow oil 0.243 g, yield 51%. ^1^H-NMR (300 MHz, DMSO-*d_6_*): 9.67 (brs, 1H), 9.52 (brs, 1H), 8.98 (s, 1H), 8.14 (s, 1H), 8.03–8.01 (m, 1H), 7.88 (s, 1H), 7.38 (d, *J* = 8.7 Hz, 2H), 7.28–7.13 (m, 7H), 7.01–6.96 (m, 1H), 6.02 (s, 1H).

#### 3.2.17. N1*-(2,4-dinitro-5-(pyridin-3-ylamino)phenyl)-*N2*-(pyridin-3-yl)benzene-1,2-diamine* (**19b**)

Compound **17** (0.215 g, 1.0 mmol) was suspended in anhydrous methanol (10 mL). Pd/C (10%, 40 mg) was added to the suspension and the mixture was hydrogenated under atmospheric pressure until the reaction mixture turned colourless. Then the Pd/C was filtered off. The filtrate was concentrated *in vacuo* to give a light yellow oil. The oil obtained was dissolved in THF (10 mL), and the solution was added **16b** (0.278 g, 1.0 mmol) and diisopropylethylamine (0.17 mL, 1.0 mmol). The reaction mixture was refluxed for another 20 h. and then cooled to room temperature The mixture was concentrated *in vacuo* and the residues were added with distilled water and filtered. The crude product was purified via chromatography (PE/EA: 2/1 to 1/2) to produce the title compound as a yellow solid 0.3 g, yield 68%. ^1^H-NMR (300 MHz, DMSO-*d_6_*): 9.72 (brs, 1H), 9.52 (brs, 1H), 8.99 (s, 1H), 8.41–8.36 (m, 1H), 8.15 (t, *J* = 1.5 Hz, 1H), 8.02 (t, *J* = 3.0 Hz, 1H), 7.85 (s, 1H), 7.64–7.60 (m, 1H), 7.35 (dd, *J* = 8.1 Hz, 4.8 Hz, 1H), 7.23 (d, *J* = 7.8 Hz, 1H), 7.17–7.14 (m, 4H), 6.94–6.88 (m, 1H), 5.92 (s, 1H).

#### 3.2.18. *(*E*)-*N*-(4-chlorophenyl)-3-(isopropylimino)-5-(pyridin-3-yl)-3,5-dihydrophenazin-2-amine* (**21a**)

Compound **19a** (0.243 g, 0.5 mmol) was suspended in glacial acetic acid (10 mL). The suspension was added with zinc powder (0.66 g) portionwise in an ice bath. After addition, the mixture was stirred at room temperature for 30 min. and then heated at 50 °C for 30 min. After being cooled to room temperature, the reaction mixture was filtered and washed with glacial acetic acid. The filtrate was concentrated *in vacuo*. The residues were added with concentrated aqueous ammonia until the mixture became basic and then the solid was filtered out, washed with water and dried to give black solid. The black solid was dissolved in anhydrous methanol and the solution was added with solution of ammonia in methanol to adjust the solution to basic. The mixture was stirred in contact with air overnight and then concentrated *in vacuo*. The residues were mixed with dioxane (5 mL) and isopropylamine (2 mL, 24 mmol). The mixture was heated at 110 °C in a sealed tube for 10 h. After being cooled to room temperature, the reaction mixture was added with water and filtered to give crude product. The crude product was purified via chromatography (PE/EA: 2/1 to EA) to produce 21 mg of the title compound as a red solid, yield 9%. Mp: 237–239 °C. ^1^H-NMR (300 MHz, DMSO-*d_6_*): 8.93 (dd, *J* = 4.8 Hz, 1.5 Hz, 1H), 8.77 (dd, *J* = 1.8 Hz, 1H), 8.62 (brs, 1H), 8.15–8.11 (m, 1H), 7.87–7.83 (m, 1H), 7.67–7.63 (m, 1H), 7.48–7.41 (m, 4H), 7.24–7.21 (m, 2H), 6.73 (s, 1H), 6.44–6.41 (m, 1H), 5.12 (s, 1H), 3.39–3.30 (m, 1H), 1.03 (d, *J* = 6.3 Hz, 6H). ^13^C-NMR (100 MHz, DMSO-*d_6_*): 151.0, 150.1, 143.0, 138.8, 137.5, 135.2, 134.7, 133.9, 131.2, 129.3, 128.2, 127.9, 126.8, 126.0, 122.9, 113.9, 88.5, 48.9, 44.8, 23.3. HRMS (*m/z*) calcd. for C_26_H_23_N_5_Cl (M+H^+^): 440.1636, found 440.1635.

#### 3.2.19. *(*E*)-3-(isopropylimino)-*N,*5-di(pyridin-3-yl)-3,5-dihydrophenazin-2-amine* (**21b**)

Compound **19b** (0.3 g, 0.7 mmol) was suspended in glacial acetic acid (5 mL). Zinc powder (1.33 g) was added portionwise to the suspension in an ice bath. After addition, the mixture was heated at 50 °C for 1 h. After being cooled to room temperature, the reaction mixture was filtered and washed with glacial acetic acid. The filtrate was concentrated *in vacuo*. The residues were added with concentrated aqueous ammonia until the mixture became basic and then the solid was filtered out, washed with water and dried to give a dark brown solid. The solid was dissolved in anhydrous methanol and the solution was added with ammonia in methanol to adjust the solution to basic. The mixture was stirred in contact with air for 7 h. and then concentrated *in vacuo*. The residues were mixed with dioxane (5 mL) and isopropylamine (0.036 mol, 3 mL). The mixture was heated at 110 °C in a sealed bomb for 10 h. After being cooled to room temperature, the reaction mixture was added with water and filtered to give crude product. The crude product was purified via chromatography (PE/EA: 2/1 to EA) to produce 40 mg of the title compound as a red solid, yield 15%. Mp: 174–175 °C. ^1^H-NMR (400 MHz, DMSO-*d_6_*): 8.92 (dd, *J* = 4.8 Hz, 1.2 Hz, 1H), 8.76 (d, *J* = 2.4 Hz, 1H), 8.62 (brs, 1H), 8.60 (d, *J* = 2.4 Hz, 1H), 8.32 (d, *J* = 4.0 Hz, 1H), 8.13–8.11 (m, 1H), 7.87–7.83 (m, 1H), 7.65–7.63 (m, 1H), 7.44 (dd, *J* = 8.0 Hz, 4.8 Hz, 1H), 7.23–7.20 (m, 2H), 6.65 (s, 1H), 6.43–6.41 (m, 1H), 5.12 (s, 1H), 3.36–3.33 (m, 1H), 1.03 (d, *J* = 6.0 Hz, 6H). ^13^C-NMR (100 MHz, DMSO-*d_6_*): 151.0, 150.1, 150.0, 144.2, 143.9, 143.6, 137.4, 136.7, 135.2, 134.7, 133.9, 131.2, 128.5, 128.1, 127.9, 126.0, 123.9, 123.0, 113.9, 98.8, 88.5, 48.9, 23.3. HRMS (*m/z*) calcd. for C_25_H_23_N_6_ (M+H^+^): 407.1978, found 407.1980.

#### 3.2.20. Synthesis of *1-[5-(3-pyridylamino)-2, 4-dinitroanilino]-2-(4-chloroanilino)benzene* (**22**)

A mixture of compound **11b** (40.3 g, 100 mmol), 3-aminopyridine (14.1 g, 150 mmol) and triethylamine (14 mL, 100 mmol) in THF (200 mL) was refluxed under nitrogen atmosphere for 28 h. About 150 mL of THF was evaporated at atmospheric pressure. CH_2_Cl_2 _was added and the solid formed was filtered and washed with CH_2_Cl_2_ to give the title compound **22** (31.2 g, 66%). ^1^H-NMR (300 MHz, DMSO-*d*_6_) *δ* 9.72 (s, 1H), 9.00 (s, 1H), 8.40–8.37 (m, 2H), 7.77 (s, 1H), 7.63–7.59 (m, 1H), 7.37–7.33 (m, 1H), 7.21–7.09 (m, 5H), 6.92–6.83 (m, 1H), 6.82–6.78 (m, 2H), 5.92 (s, 1H). ESI/MS (*m/z*): 477 [M+H]^+^.

#### 3.2.21. Synthesis of *2-(3-pyridylamino)-5-(4-chlorophenyl)-3-imino-3,5-dihydrophenazine* (**24**)

Zinc powder (71 g, 1.1 mol) was added portionwise to a mixture of compound **22** (28.6 g, 60 mmol) in glacial acetic acid (150 mL) cooled in an ice water bath. The mixture was stirred until the colour turned to light green, and then filtered, washed with glacial acetic acid and anhydrous methanol. The filtrate was concentrated and the residue was treated with water and adjusted to alkaline with ammonia. The solid formed was filtered, washed with water and then dissolved in anhydrous methanol. The methanol solution was stirred in contact with air overnight. The solid formed was filtered to produce 23.1 g of crude **24** which was used in the next step without further purification.

#### 3.2.22. Synthesis of *5-(4-chlorophenyl)-3-isopropylimino-2-(3-pyridylamino)-3,5-dihydrophenazine* (**25**)

A mixture of compound **24** (23.1 g), isopropylamine (100 mL, 1.2 mol) and dioxane (120 mL) was heated in a sealed tube at 110 °C for 7 h. After cooling, the reaction mixture was treated with water and the solid formed was filtered off and washed with water. The crude product was purified by column chromatography (eluted with hexane/ethyl acetate: 2:1) to produce the title compound **25** as a red solid (11.2 g, 44%). M.p. 194–196 °C. ^1^H-NMR (300 MHz, DMSO-*d*_6_) *δ* 8.63 (brs, 1H), 8.61 (d, *J* = 2.4 Hz, 1H), 8.32 (dd, *J* = 4.8, 1.2 Hz, 1H), 7.90–7.84 (m, 3H), 7.65–7.58 (m, 3H), 7.25–7.18 (m, 2H), 6.66 (s, 1H), 6.47–6.44 (m, 1H), 5.76 (s, 1H), 3.43–3.35 (m, 1H), 1.06 (d, *J* = 6.3 Hz, 6H). ^13^C-NMR (100 MHz, DMSO-*d*_6_) *δ* 150.2, 149.9, 144.1, 143.8, 143.5, 136.8, 135.8, 135.3, 134.4, 131.6, 131.1, 130.9, 128.3, 128.0, 127.8, 123.9, 122.9, 114.1, 98.8, 88.3, 48.9, 23.3. HRMS (ESI-TOF^+^): [M+H]^+^ calcd. for C_26_H_23_ClN_5_: 440.1641; found: 440.1643.

### 3.3. Biological Evaluation

*In* *vitro* and *in vivo* anti-tuberculosis activities were determined using our routine methods [9].

## 4. Conclusions

In summary, we have performed a systematic SAR and SLR investigation by synthesizing and evaluating a variety of analogues of clofazimine with modifications at various positions. The study revealed that the central tricyclic ring system is the pharmacophore of the molecule and is important for anti-tuberculosis activity. Attempts to simplify this pharmacophore led to compounds with reduced antimycobacterial activity. The two phenyl rings appended to the C2 and N5 positions can be replaced by a pyridyl ring. Replacement of the phenyl group attached to the C2 position by a pyridyl group leads to compounds with not only improved *in vitro* and *in vivo* anti-tuberculosis activity, but also favourable pharmacokinetic profiles with reduced skin pigmentation potential. 

## References

[1] World Health Organization (WHO) (2009). Global Tuberculosis Control 2009: Epidemiology, Strategy, Financing.

[2] Chan E.D., Iseman M.D. (2002). Current medical treatment for tuberculosis. Br. Med. J..

[3] Perri G.D., Bonora S. (2004). Which agents should we use for the treatment of multidrug-resistant *Mycobacterium tuberculosis?*. J. Antimicrob. Chemother..

[4] Barry V.C., Buggle K. (1960). Absorption, distribution and retention of the riminocompounds in the experimental animal. Ir. J. Med. Sci..

[5] Barry V.C., Conalty M.L. (1965). The antimycobacterial activity of B663. Lepr. Rev..

[6] Reddy V.M., Nadadhur G. (1996). Antituberculosis activities of clofazimine and its new analogs B4154 and B4157. Antimicrob. Agents Chemother..

[7] Reddy V.M., O’Sullivan J.F., Gangadharam P.R. (1999). Antimycobacterial activities of riminophenazines. J. Antimicrob. Chemother..

[8] O’Connor R., O’Sullivan J.F. (1995). The pharmacology, metabolism, and chemistry of clofazimine. Drug Meta. Rev..

[9] Lu Y., Zheng M., Wang B. (2011). Clofazimine analogs with efficacy against experimental tuberculosis and reduced potential for accumulation. Antimicrob. Agents Chemother..

